# Artificial Intelligence-Based Prediction of Oroantral Communication after Tooth Extraction Utilizing Preoperative Panoramic Radiography

**DOI:** 10.3390/diagnostics12061406

**Published:** 2022-06-06

**Authors:** Andreas Vollmer, Babak Saravi, Michael Vollmer, Gernot Michael Lang, Anton Straub, Roman C. Brands, Alexander Kübler, Sebastian Gubik, Stefan Hartmann

**Affiliations:** 1 Department of Oral and Maxillofacial Plastic Surgery, University Hospital of Würzburg, 97070 Würzburg, Germany; straub_a@ukw.de (A.S.); brands_r@ukw.de (R.C.B.); kuebler_a@ukw.de (A.K.); gubik_s@ukw.de (S.G.); hartmann_s2@ukw.de (S.H.); 2 Department of Orthopedics and Trauma Surgery, Medical Centre-Albert-Ludwigs-University of Freiburg, Faculty of Medicine, Albert-Ludwigs-University of Freiburg, 79106 Freiburg, Germany; babak.saravi@jupiter.uni-freiburg.de (B.S.); gernot.michael.lang@uniklinik-freiburg.de (G.M.L.); 3 Department of Oral and Maxillofacial Surgery, Tuebingen University Hospital, Osianderstrasse 2-8, 72076 Tuebingen, Germany; michael.vollmer@med.uni-tuebingen.de

**Keywords:** artificial intelligence, deep learning, X-ray, tooth extraction, oroantral fistula, operative planning

## Abstract

Oroantral communication (OAC) is a common complication after tooth extraction of upper molars. Profound preoperative panoramic radiography analysis might potentially help predict OAC following tooth extraction. In this exploratory study, we evaluated n = 300 consecutive cases (100 OAC and 200 controls) and trained five machine learning algorithms (VGG16, InceptionV3, MobileNetV2, EfficientNet, and ResNet50) to predict OAC versus non-OAC (binary classification task) from the input images. Further, four oral and maxillofacial experts evaluated the respective panoramic radiography and determined performance metrics (accuracy, area under the curve (AUC), precision, recall, F1-score, and receiver operating characteristics curve) of all diagnostic approaches. Cohen’s kappa was used to evaluate the agreement between expert evaluations. The deep learning algorithms reached high specificity (highest specificity 100% for InceptionV3) but low sensitivity (highest sensitivity 42.86% for MobileNetV2). The AUCs from VGG16, InceptionV3, MobileNetV2, EfficientNet, and ResNet50 were 0.53, 0.60, 0.67, 0.51, and 0.56, respectively. Expert 1–4 reached an AUC of 0.550, 0.629, 0.500, and 0.579, respectively. The specificity of the expert evaluations ranged from 51.74% to 95.02%, whereas sensitivity ranged from 14.14% to 59.60%. Cohen’s kappa revealed a poor agreement for the oral and maxillofacial expert evaluations (Cohen’s kappa: 0.1285). Overall, present data indicate that OAC cannot be sufficiently predicted from preoperative panoramic radiography. The false-negative rate, i.e., the rate of positive cases (OAC) missed by the deep learning algorithms, ranged from 57.14% to 95.24%. Surgeons should not solely rely on panoramic radiography when evaluating the probability of OAC occurrence. Clinical testing of OAC is warranted after each upper-molar tooth extraction.

## 1. Introduction

**Figure 1:**
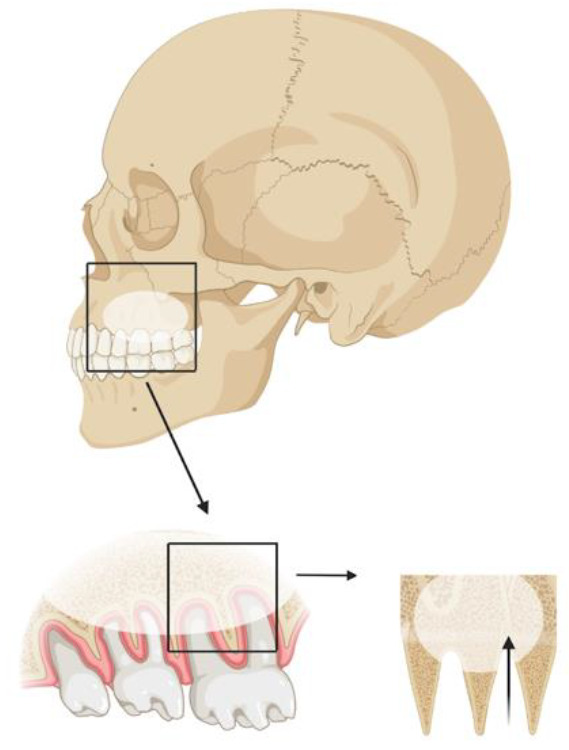
Illustration of the relationship between upper molars and the oroantral regions. Upper molar tooth extraction can lead to a perforation of the maxillary sinus floor and subsequent communication of the oral cavity with the maxillary sinus.

When teeth are surgically removed in the maxilla, the opening of the maxillary sinus is a relevant complication, especially in the posterior region. Recent studies indicate that surgical removal of the upper third molar in the maxilla may cause maxillary sinus opening in up to 13% of cases, whereas completely displaced teeth may further increase the prevalence to up to 25% [1]. Usually, primary treatments cannot prevent oroantral communication (OAC). More invasive surgical interventions than novel, less invasive ones, for example, are associated with a higher likelihood of complications [2,3]. An illustration of the relationship between upper molars and the oroantral region is shown in Figure 1. The maxillary sinus can have variable anatomy due to maxillary sinus septa, temporary mucosal swelling, previous operations (Caldwell–Luc operation), or tumors [4]. Two-dimensional radiographic imaging is the standard imaging for routine extraction of maxillary teeth [5]. Panoramic radiography is the most widely used imaging modality for common oral surgical procedures. In addition to the general overview of the maxilla and mandible in a 2D X-ray/panoramic radiography, it is also characterized by its high availability, low radiation exposure, and low cost compared to 3D cone beam computer tomography [6,7]. Surgical intervention is required when the mucosal perforation exceeds 3 mm [5]. To be able to treat this complication, preoperative planning is necessary, such as planning the incision to be able to form a possible mucoperiosteal flap [8]. Simple closures with a single suture are possible but carry a high risk of complications [9]. Preoperative risk stratification algorithms could help lower the possible postoperative complications associated with OAC by utilizing them in alert-like systems for patients at risk in clinics.

In 1978, mathematician Richard Bellman defined artificial intelligence (AI) as the automation of activities associated with human thinking skills, such as learning, decision making, and problem solving [10]. A clinical decision-support system is a computer algorithm developed to support clinical decision making in healthcare systems. This process involves processing a wide variety of medical data points necessary or valuable for interpretation [11,12]. As a branch of artificial intelligence, machine learning uses statistical learning algorithms to create systems that learn and enhance on their own without being explicitly programmed. The concept of “deep learning” is an applied machine learning method based on how the human brain filters information and learns from examples. Filtering input data through layers enables a computer model to anticipate and classify information. The term “convolutional neural networks” refers to artificial neural networks commonly applied to medical image prediction and classification. Essentially, it is a deep learning algorithm that takes an image as input and assigns weights/biases to specific characteristics and objects in the image in order to distinguish between them. CNNs are composed of many hidden layers, such as convolutional layers, pooling layers, fully connected layers, and normalizing layers. A ConvNet is designed to mimic the organization of the visual cortex and the pattern of connectivity of the neurons in the human brain [13]. In dentistry, interest in this area of research has increased significantly in recent years [14]. In a systematic review by Khanagar et al. (2021), many areas of application of AI in dentistry have already been identified [14]. The studies included in this systematic review were mainly concerned with the application of AI for the detection and diagnosis of dental caries and other oral pathologies. Here, the algorithms reached satisfying diagnostic accuracy.

A high predictive probability for sinusitis of the maxillary sinus has already been described [15]. As compared with experienced clinicians, at least a comparable level of sensitivity and specificity has been achieved [16]. Artificial intelligence is a beneficial tool to provide adequate guidance to the practitioner in case no other three-dimensional imaging is available. To the best of our knowledge, the AI-based predictive accuracy of panoramic radiography for maxillary sinus perforation after tooth extraction has not yet been described. Thus, we sought to evaluate several deep learning models for the prediction of OAC after tooth extraction utilizing preoperative panoramic radiography and compare the diagnostic accuracy with the accuracy obtained from human experts’ evaluations. The overall aim of this exploratory study is to evaluate whether the anatomical situation found in panoramic radiography can predict OAC reliably after tooth extractions. Generally, we aimed to (1) assess the feasibility of OAC prediction from preoperative panoramic radiography utilizing multiple deep learning algorithms; (2) evaluate the feasibility of OAC prediction from expert evaluations; and (3) assess whether there are differences in diagnostic metrics for expert evaluations and deep learning algorithms regarding the OAC predictions.

## 2. Materials and Methods

### 2.1. Study Design

The examination is conducted in accordance with the Declaration of Helsinki and the Professional Code of Conduct for Physicians of the Bavarian Medical Association in the respective current versions. Although informed consent is regarded as a requirement for research purposes according to the Declaration of Helsinki and the Professional Code of Conduct for Physicians of the Bavarian Medical Association in the respective current versions, the ethics committee waived the need for informed consent in the present study due to the anonymization of X-ray data. All consecutive patients examined from 2010 through 2020 at the University Hospital Würzburg with indications of tooth extraction in the posterior region of the upper jaw were included in this study. Exclusion criteria were malignant diseases in the surgical area, fractures in the surgical site, syndromal anatomical variants, inflammation process on the root tip, and chronic/pre-existing OAC. In total, 300 patients with extracted teeth were included consecutively. The study was reviewed by the Ethics Committee of the University of Würzburg and approved under the authentication number 2022011702.

The data were acquired in the data management system of the University Hospital of Würzburg. Patients who had a tooth extraction in the maxillary posterior region between 2010 and 2020 were screened. These patients were explicitly selected based on ICD codes. The respective operation report was reviewed in detail for the group of patients who had an OAC after tooth extraction. The preoperative panoramic radiography was extracted only in the case that OAC could be determined clinically with various examinations. The panoramic radiography was extracted as a completely anonymized image file. For the control group, patients who had an extraction in the maxillary posterior region were searched and allocated to the control group after reviewing the surgical report, in which OAC was excluded and/or not diagnosed. The extraction of the radiograph was performed in the same way as described above. Overall, 100 consecutive cases with similar image and positioning quality in the OAC group (from 2010 to 2020) and 200 cases in the control group were collected for data analysis.

### 2.2. Expert Evaluations

In order to evaluate and compare the results of the deep learning algorithms, a comparative analysis was carried out by four oral and maxillofacial clinicians. A sequence of a total of 300 randomly arranged panoramic radiography images was produced. This sequence included a total of 100 images with a postoperative OAC and 200 images without this complication. The examiners were asked to decide from the preoperative panoramic radiography whether or not postoperative OAC occurs after the extraction of teeth in the maxillary posterior region (binary code: 1 for OAC and 0 for non-OAC). The diagnostic performance was determined for the respective practitioners and compared with the results of the deep learning algorithms.

### 2.3. Convolutional Neural Networks

The original images were taken utilizing multiple panoramic imaging devices. Images were randomly split into a train, test, and validation sets (60%, 20%, 20%). The validation data comprised the dataset used during training to check the outcome and adopt the model structure/hyperparameters. The test data comprised the hold-out dataset that was not used until the training process was finished to evaluate metrics. Then, we rescaled (224 × 224) all dataset images and pre-processed the train dataset images by applying data augmentation techniques (rotation range of ±30 degrees, horizontal flipping, brightness of 20–80%). Image augmentation was used to reduce overfitting and improve generalization. The region of interest was set manually by one surgeon to define the maxilla and the sinus area. We then utilized multiple supervised pre-trained deep learning models to classify the two study classes OAC versus non-OAC. For this, we applied five deep learning models (VGG16, ResNet50, Inceptionv3, EfficientNet, and MobileNetV2) to solve the classification problem. The algorithms’ structure and the code are available in the data availability section. The models were frozen in the way that we used the basic models and made changes to the final layer only, as these models were designed to handle multiple classes, whereas we needed to solve a binary classification problem. For this, we made the layer non-trainable and built a last fully connected layer. Overall, we flattened the output of our base model to one dimension, added a fully connected layer with hidden units and ReLU activation, used a dropout rate, and added a final fully connected sigmoid layer. The specific characteristics of the models, including each layer, are shown in the code available in the data availability section. We used the RMSProp Optimizer (VGG16, InceptionV2, EfficientNet), SGD optimizer (ResNet50), or Adam optimizer (MobileNetV2) with a learning rate of 0.0001 and binary cross-entropy for loss evaluation. Steps per epoch were calculated as the sample size for the training set divided (using the integer division operator) by the batch size, where the batch size was 10. Models were trained for 10 epochs. We did not use a grid search, random search, or Bayesian optimization for hyperparameter tuning but used a manual search to adjust the parameters until the best metrics were obtained. Grid search and manual search are the most widely used strategies for hyper-parameter optimization [17]. Hyperparameter tuning using fine-tuning algorithms was intended to be applied to improve models more precisely in the case where an AUC over 0.75 could be reached for any model. In case no evidence was found that models were suitable to reach higher accuracies, we decided not to perform further hyperparameter tunings in a resource-oriented way, as these fine-tuning techniques are more intended to build precise models to classification tasks than to explore the feasibility/exploratory approach of whether a reliable classification is possible or not.

To evaluate each model’s performance, accuracy, precision, recall, F1 score, and AUC were calculated. Accuracy is a metric used in classification problems to determine the percentage of accurate predictions. Precision is the ratio of true positives to true positives and false positives. The recall consists of the proportion of true positives to true positives and false negatives. An F1 score is derived by dividing precision and recall by (2 × precision + recall)/(precision + recall), while the AUC represents the area under the receiver operating characteristics (ROC) curve. To evaluate the clinical usability of AI, the results of panoramic radiography reads by AI and four oral and maxillofacial surgery specialists were compared. The diagnostic performance was assessed using the AUC, sensitivity, and specificity metrics. Agreement for expert evaluations was assessed with Cohen’s kappa statistics. Algorithms were built and evaluated in Python using the OpenCV, NumPy, Pillow, Seaborn, Matplotlib, TensorFlow, Keras, and scikit-learn libraries. The hardware and software environment specifications were as follows:

- CPU: AMD Ryzen 9 5950X 16-Core Processor;
- RAM: 64 GB;
- GPU: NVIDIA Geforce RTX 3090 (24 GB GDDR6X RAM);
- Python version: 3.10.4 (64-bit);
- OS: Windows 10.

Statistical analyses were conducted in Python, Stata Statistical Software Release 15 (StataCorp. 2011, College Station, TX, USA), and SPSS v26 (IBM, Armonk, NY, USA). Figure 1 was created with BioRender.com software (BioRender, Toronto, ON, Canada).

## 3. Results

### 3.1. Convolutional Neural Network Performance

According to the accuracy and area under the curve (AUC) measure, the best-performing models were MobileNetV2 and IncepionV3. The accuracy, AUC, precision, recall, and F1 score for the MobileNetV2 model were 0.74, 0.67, 0.75, 0.43, and 0.55, respectively (Table 1). The accuracy, AUC, precision, recall, and F1 score for the InceptionV3 model were 0.70, 0.60, 1.00, 0.19, and 0.32, respectively.

The confusion matrices and metrics of each model performed on the hold-out dataset (validation dataset) can be found in the data availability section. The specificity ranged from 0.8611 to 1.0000, with the highest specificity reached by the InceptionV3 model. The sensitivity ranged from 0.0476 to 0.4286, with the highest sensitivity reached by the MobileNetV2 model. The sensitivities from the EfficientNet, InceptionV3, MobileNetV2, ResNet50, and VGG16 were 0.0476, 0.1905, 0.4286, 0.0476, and 0.1429, respectively. The specificities from the EfficientNet, InceptionV3, MobileNetV2, ResNet50, and VGG16 were 0.9722, 1.0000, 0.9167, 0.8611, and 0.9167, respectively. The false-negative rate, i.e., the rate of true-positive cases (OAC) that were missed by the algorithms, ranged from 57.14% (MobileNetV2) to 95.24% (EfficientNet and ResNet50).

### 3.2. Expert Evaluations

Table 2 shows the performance metrics for each of the four expert evaluations. The area under the curve (AUC) ranged from 0.5458 to 0.7059. The specificity ranged from 51.74% to 95.02%, whereas the sensitivity ranged from 14.14% to 59.60%. Cohen’s kappa exhibited a poor agreement for the oral and maxillofacial expert evaluations (Cohen’s kappa: 0.1285).

**Figure 2:**
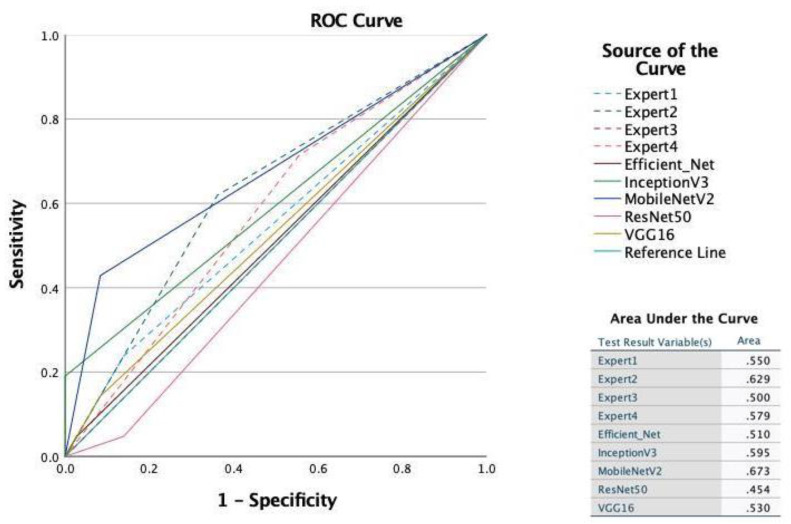
Receiver operating characteristic (ROC) curves and area under the ROC curves for all deep learning models and examiners.

The comparison of all ROC curves and AUC is shown in Figure 2 The deep learning model MobileNetV2 reached the highest AUC (AUC: 0.673), followed by a human expert (expert 2; AUC: 0.629).

## 4. Discussion

The present study sought to evaluate the feasibility of OAC prediction after upper-molar tooth extraction utilizing preoperative panoramic radiography. The results showed that although the MobileNetV2 algorithm and one expert reached an AUC of 0.673 and 0.629, respectively, the overall predictability of OAC from panoramic radiography was low. The false-negative rate, i.e., the rate of positive cases (OAC) missed by the deep learning algorithms, ranged from 57.14% to 95.24%. Further, there was a poor agreement for the oral and maxillofacial expert evaluations (Cohen’s kappa: 0.1285).

Due to the fact that there are no comparable predictive studies currently available, it is not possible to compare our diagnostic metrics with others for the prediction of OAC after upper-molar tooth extraction. Using two data sets, one study compared AI-based and human examiner-based evaluations of inflammatory processes in the maxillary sinus from panoramic radiography [16]. The AI-based models achieved an AUC of 0.93 and 0.88 compared to the radiologist with 0.83 and 0.89. For predicting the postoperative injury of the inferior alveolar nerve from panoramic radiography, a systematic review of current evidence showed that sensitivity ranges from 0.06 to 0.49, and specificity ranges from 0.42 to 0.89, which is in accordance with our deep learning results for OAC [6]. These findings were also comparable to our expert evaluations for OAC although the agreement between the experts was low. We could not find a general superiority of the AI-based algorithms compared to the expert evaluations, as described before for panoramic radiography predictions; however, one deep learning algorithm reached the highest AUC [6,16]. This finding may be due to the fact that the information available in panoramic radiography was not sufficient to detect patterns on the basis of which an AI would be able to predict OAC reliably. One systematic review evaluated several risk assessment studies assessing the risk of OAC based on clinical data, panoramic radiography, or cone-beam computer tomography (CBCT) utilizing statistical models. The authors concluded that panoramic radiographies are not reliable for assessing risk factors for OAC compared to CBCT based on current evidence [5]. We could confirm this finding by applying multiple deep-learning algorithms and letting experts evaluate the preoperative panoramic radiography.

Two-dimensional images (panoramic radiography) are not able to reflect the three-dimensional anatomical situation of molar roots. Bouquet et al. were able to show that in panoramic radiography, the root of the tooth appeared to protrude into the maxillary sinus, whereas in three-dimensional imaging (CBCT), there was no contact and thus no anatomical relationship [18]. Teeth can appear more inclined than they are in panoramic radiography [18]. This finding can be explained by the fact that deformations can occur when projecting a volume onto a flat surface. Such deformations are not expected in a 3D image [18]. It must also be borne in mind that in the majority of cases, the spatial development of the maxillary sinus is buccal to the roots of the maxillary molars [18,19]. For this reason, the analysis of the more palatal/distal tooth part seems to be less relevant for the chosen question of perforation of the maxillary sinus. Furthermore, no information is available regarding the number of roots. Iwata et al. showed that single-rooted teeth had a higher incidence of oroantral connections than multi-rooted teeth [20].

Using a defined classification (Archer classification, inclination, and root sinus classification), it has been shown that the positional relationship of maxillary molars to the maxillary sinus or their neighboring teeth can predict the probability of OAC [20]. In addition, other factors such as treatment components (incision, bone removal, maxillary tuber fractures, and extensive bleeding) correlate significantly with the likelihood of OAC [20]. The multifactorial genesis makes a prediction using 2D imaging difficult even with reliable classification systems. If the positional relationship or number of roots is unclear, 3D imaging is a helpful tool [18]. For AI-based prediction models, it is therefore difficult to reliably predict the occurrence of OAC based only on 2D imaging. It remains to be verified whether prediction with 3D imaging, for example, 3D magnet resonance tomography for soft tissue illustrations, can produce better results because of the additional information processed with an AI approach in specific classification tasks [21]. In the expert evaluations, we also showed that only low agreement could be identified between experts, indicating that 2D imaging is also not sufficient to predict OAC from the clinical perspective.

At present, deep learning methods are still being developed. An important advantage of convolutional neural networks is their ability to rapidly develop a feature extraction model, which is not overly concerned with the effectiveness of some features. It is, however, difficult to compare and explain performance. MobileNetV2 is one of the most popular deep learning methods that are widely used today since it has one of the most lightweight network architectures. This model showed the best performance in our study. MobileNetV1 introduced depth-wise separable convolution, which dramatically reduced the network’s complexity costs and model size, making it suitable for low-processing devices, such as smartphones. In MobileNetV2, a better module with the inverted residual structure, is introduced. It eliminates non-linearities in narrow layers. In addition to achieving state-of-the-art performances for feature extraction, MobileNetV2 also achieves advanced results for object detection and semantic segmentation [22]. In general, MobileNetV2 is very similar to the original MobileNet, with the exception that it uses a novel layer module called the inverted residual with linear bottleneck, which reduces the memory requirement for processing since it has fewer parameters than the original MobileNet. As a result, the MobileNet V2 is less prone to overfitting. The proposed method uses MobileNetV2 as the basis for the transfer learning process. Due to the lightweight network architecture, the developed model can be implemented more quickly in clinical settings or mobile devices, making it more practical for use in clinical settings. Additionally, we included MobileNetV2 because a recent study showed that it is possible to perform classification tasks from panoramic radiographs with MobileNetV2 achieving higher accuracy than has been seen in the past, for instance, in classifying caries in the third molars [23]. Apostolopoulos et al. used VGG19 and MobileNetV2 to perform feature extraction on X-ray images and found that MobileNetV2 performed better than VGG19 in terms of specificity [24]. As a result, they believe that MobileNetV2 is the most robust model for specific classification tasks and data samples. In general, more research needs to be undertaken in order to evaluate why MobileNetV2 outperforms other methods in various settings. Notably, there might also be other image classification algorithms that could outperform the included models, such as artificial neural networks based on the successive geometric transformations model (SGTM) [25]. Several studies involving CNN in orthopedics, oncology, ophthalmology, and neurosurgery have been cited in the *PubMed* database since 2013. In 2017, Miki et al. published one of the first reports using CNNs with cone-beam computed tomography in the dental field [26]. CNN has been used in recent publications in cariology, periodontology, and endodontics as well as practical applications in clinics that are to be exploited in the near future [27,28,29]. Recent research describes a method to identify teeth using orthopantomography and registering them using simple CNNs that can help dentists in filling out dental charts more quickly and efficiently [30]. Other researchers developed a method of calculating age utilizing global fuzzy segmentation and local feature extraction based upon a projection-based feature transformation with a deep CNN model designed for molar classification [31]. In a scoping study on CNN applications in dental image diagnostics, it was observed that CNNs could be utilized in diagnostic-assistance systems in the dental arena [32]. At present, the implementation of CNN technology is challenging for dentists. It is expected that the generalization of such technology will be made easier through the development of improved algorithms. Previously, discriminant handcrafted features (e.g., histograms of oriented gradients features or local binary patterns features) dominated digital image analysis, but recent advances in deep learning algorithms have displaced the handcrafted approach, allowing automated image analysis. Convolutional neural networks are a type of deep learning algorithm that has become a workhorse. In recent data challenges for medical image analysis, all of the top-ranked teams used CNN. Except for one team, the top ten ranked solutions in the CAMELYON17 challenge used CNN for automatic detection and classification [33]. Shi et al. showed that the characteristics recovered via deep learning are superior to those extracted from handmade approaches [34]. In practice, however, deep learning algorithms such as CNN require a considerable quantity of training data under ideal conditions, resulting in a data-scarcity problem. A number of obstacles, such as the scarcity of expert-annotated data sets and the small size of medical cohorts, are well-known. Several studies have attempted to solve this problem by utilizing transfer learning or domain adaptation [35]. These approaches try to produce a high performance on target activities by applying knowledge learned from source tasks. Recent studies of transfer learning approaches from a data and model perspective were reviewed in 2020 by Zhuang et al. [36]. Researchers are increasingly interested in unsupervised transfer learning, an emerging academic subject. In their review of unsupervised deep domain adaptations, Wilson and Cook [37] examined a large number of articles. The use of generative adversarial networks-based frameworks has gained momentum recently [38], with Domain Adversarial Neural Network (DANN) being particularly promising [39]. A number of other methods have also been utilized for unsupervised transfer learning, including multiple kernel active learning [40] and collaborative unsupervised methods [41].

The study is associated with strengths and limitations. To the best of our knowledge, it is the first study evaluating the prediction of OAC utilizing both AI-based and expert-based evaluations of preoperative panoramic radiography. Thus, it contributes to the existing evidence, which solely applied statistical modeling (i.e., regression models) to evaluate risk factors for OAC. Further, the presented algorithms and dataset can be used to expand the methodology, compare diagnostic metrics with 3D assessment metrics, and perform external validations. However, there are also limitations associated with the present study. Unknown confounding factors due to the nature of retrospective analysis must be considered. In retrospective studies, it must be taken into account that small OAC may have occurred and were not documented in the patient information system, as this did not result in any additional need for intervention. Hence, although we accurately checked the available surgical reports to ensure whether OAC occurred or not, there might be misallocations. Thus, it should be noted that the control group included cases that were not assigned to the intervention group due to the lack of documentation in cases of low clinical suspicion, small OAC not worthy of treatment, or OAC that had occurred but were not documented. This might bias the allocation process. A more precise allocation would be possible with a prospective study design with standardized clinical testing algorithms for OAC. Overall, external validation utilizing prospective datasets is warranted. Another limitation is the determination of the ROI in our study. We decided to include a rather larger ROI to evaluate whether shapes of the sinus or adjacent structures are related to OAC. This was based on a previous study showing that the Archer and Root Sinus classification of teeth impaction is significantly associated with OAC [20]. As both classifications focus not only on the extracted teeth but also on adjacent structures, we decided to include a larger ROI. In our subsequent study, including larger sample size, we limited the ROI to the sinus area to evaluate whether the automatized classification of Archer and RS classes would be possible (unpublished data). In addition, here, we did not find evidence that panoramic radiography is feasible for this classification task, which is also in accordance with the expert evaluations. Although we included an extensive period to extract all images in our institution, the number of OAC cases might still be small, limiting the capabilities of deep learning algorithms to reliably learn the features from the dataset that can predict OAC, potentially reflecting the low sensitivity obtained from our algorithms. Sample size calculations for image classifications are known to require more than 1000 images per class for accurate predictions. However, this is often not possible in monocentric studies coming from surgical departments, as also shown in a recent systematic review assessing whether studies to date have performed sample size calculations for deep learning purposes in the literature [42]. These sample-size calculations might be more beneficial if there is evidence in an initial dataset analysis showing that classification is accurate and feasible from the dataset. A subsequent sample-size calculation can further improve future research models to a specific degree although studies have shown that sample size also affects the robustness of neural networks [43]. Another common mistake is to use the same data sets for validation and training. To avoid this bias, we separated the dataset into a training, testing, and validation sets, limiting the size of the training dataset further [44,45]. Nevertheless, the present study was the first feasibility study to evaluate whether multi-center studies would be beneficial in assessing the study question. As we did not find convincing evidence that panoramic radiography can predict OAC, our approach might have saved research resources associated with multi-center evaluations. Notably, the predictions of the algorithms are exclusively based on panoramic radiography. In this case, the practitioner’s clinical decision-making process, which is carried out by considering all additional clinical data (i.e., clinical examinations, the extent of surgical invasiveness), cannot be fully simulated by the AI algorithms [46]. In addition, binary classification by human experts might not be as accurate as Likert-like scales or visual analogue scales, where expert decisions might be better reflected. This approach would also be more comparable with the algorithms that provide the probability metrics. Notably, we used the whole dataset for expert evaluations, which might be a discussion point, as this strategy limit the comparability with the metrics obtained from the hold-out dataset of the deep learning models. For metrics evaluations in deep learning, we used the metrics of the test dataset (hold-out dataset) because the same dataset to evaluate the model metrics should not be used as the dataset used to train (train dataset) and fine-tune the model (validation dataset). This approach was not necessary for the expert evaluations, thus justifying the use of the whole dataset for the evaluation process to evaluate whether experts are able to detect OAC from the dataset. Furthermore, the comparison of metrics between institutions may be limited due to different radiography protocols [44]. In addition, the surgical approach and the individual experience of the practitioner (i.e., learning curves) cannot be fully considered in prediction studies trying to predict OAC from panoramic radiography. Although prospective studies could adjust their study designs to evaluate data from solely one surgeon with a single technique extracting wisdom teeth, this seems not feasible considering that large datasets are required for deep learning evaluations. It is an inherent limitation of artificial intelligence-based algorithms based on only one data modality to lack multi-perspectivity when predicting images. Multi-input-mixed data hybrid models could help to improve the predictive capacities in the future [12]. In summary, the decision making based on AI algorithms remains complex and is beyond the practitioner’s control [47,48]. Thus, clinical applicability may be limited. However, our primary aim was not to evaluate the algorithms as potential alert-like systems in clinics that can help to screen patients at risk for OAC but to generally evaluate the feasibility of OAC prediction based on preoperative panoramic radiography. Although such alert-like systems may be interesting in clinics, the authors recommend testing clinically whether an OAC has occurred after each extraction. Various options have been established for clinical testing. Starting with the least invasive test, the Valsalva test puts pressure on the maxillary sinus and, therefore, a possible OAC. The escaping air can be detected by air bubbles, a whistling sound, or a fogging mirror. However, this test can be falsely negative if mucous membranes are obstructed. Blunt probing and the insertion of objects impermeable to X-rays are not recommended because of their invasiveness and the possibility of germs spreading into the maxillary sinus [49]. Although the aforementioned clinical tests have limitations, they might be the easiest, fastest, and most accurate option currently available when considering the available evidence and our results.

Final clinical decisions should be made considering all aspects that potentially affect patients and can only be made by the practitioner. Supporting this decision-making process with the objective perspective of an AI-based approach may improve the quality of treatment. However, in the context of the present results, both experts and deep learning algorithms were not able to predict OAC reliably from patients’ panoramic radiography.

## 5. Conclusions

Whether preoperative panoramic radiography information can help predict OAC after a tooth extraction is currently unknown. The results showed that although the MobileNetV2 algorithm and one expert reached an AUC of 0.673 and 0.629, respectively, the overall feasibility of OAC prediction from panoramic radiography was low. The false-negative rate, i.e., the rate of positive cases (OAC) missed by the deep learning algorithms, ranged from 57.14% to 95.24%. Further, there was a poor agreement for the oral and maxillofacial expert evaluations (Cohen’s kappa: 0.1285). AI approaches utilized in the present work seem to be not feasible in predicting OAC based on the results shown. However, larger sample sizes, modification of the region of interest, and the inclusion of other algorithms could help to improve the knowledge presented with the work. Surgeons should not solely rely on panoramic radiography when evaluating the probability of OAC occurrence. Clinical testing of OAC is warranted after each upper-molar tooth extraction.

## Figures and Tables

**Table 1:** Model performance of the convolutional neural networks. Values show the metrics for the independent test dataset (hold-out dataset).

Algorithm	Accuracy	AUC	Precision	Recall	F1-Score
VGG16	0.63	0.53	0.50	0.14	0.22
MobileNetV2	0.74	0.67	0.75	0.43	0.55
InceptionV3	0.70	0.60	1.00	0.19	0.32
ResNet50	0.56	0.45	0.17	0.05	0.07
EfficientNet	0.63	0.51	0.50	0.05	0.09

Precision, TP/(TP + FP); Recall, TP/(TP + FN); F1 score, 2 × (recall × precision)/(recall + precision); AUC, area under the curve; Accuracy, (TP + TN)/(TP + TN + FP + FN).

**Table 2:** Detailed report of examiners (n = 300). AUC: area under the receiver operating characteristic (ROC) curve.

Observer	Sensitivity	Specificity	Correctly Classified	AUC
1	14.14	95.02	68.33	0.5458
2	59.60	81.59	74.33	0.7059
3	34.69	76.12	62.54	0.5541
4	68.69	51.74	57.33	0.6021

## Data Availability

Algorithm metrics are provided in Supplementary File S1. The raw images are anonymized and available from the corresponding author on reasonable request. Example images for pre-processing are shown in Supplementary File S2. The python code and deep learning algorithm structures are available from: https://github.com/Freiburg-AI-Research (accessed on 6 January 2022).

## Supplementary Materials

The following supporting information can be downloaded at: https://www.mdpi.com/article/10.3390/diagnostics12061406/s1, Figure S1: Confusion matrix, model performance measures, and receiver operating characteristic (ROC) curve for the EfficientNet algorithm. Precision: TP/(TP + FN); Recall: TP/(TP + FN); F1 score: 2*(recall*precision)/(recall + precision); support: actual occurrence of the class in the dataset. Values are showing the metrics for the independent test dataset (hold-out dataset); Figure S2: Confusion matrix, model performance measures and receiver operating characteristic (ROC) curve for the InceptionV3 algorithm. Precision: TP/(TP + FN); Recall: TP/(TP + FN); F1 score: 2*(recall*precision)/(recall + precision); support: actual occurrence of the class in the dataset. Values are showing the metrics for the independent test dataset (hold-out dataset); Figure S3: Confusion matrix, model performance measures and receiver operating characteristic (ROC) curve for the MobileNetV2 algorithm. Precision: TP/(TP + FN); Recall: TP/(TP + FN); F1 score: 2*(recall*precision)/(recall + precision); support: actual occurrence of the class in the dataset. Values are showing the metrics for the independent test dataset (hold-out dataset); Figure S4: Confusion matrix, model performance measures and receiver operating characteristic (ROC) curve for the ResNet50 algorithm. Precision: TP/(TP + FN); Recall: TP/(TP + FN); F1 score: 2*(recall*precision)/(recall + precision); support: actual occurrence of the class in the dataset. Values are showing the metrics for the independent test dataset (hold-out dataset); Figure S5: Confusion matrix, model performance measures, and receiver operating characteristic (ROC) curve for the VGG16 algorithm. Precision: TP/(TP + FN); Recall: TP/(TP + FN); F1 score: 2*(recall*precision)/(recall + precision); support: actual occurrence of the class in the dataset. Values are showing the metrics for the independent test dataset (hold-out dataset); Figure S6: Example images from the whole data set.
