# Supplementary material for: Artificial Intelligence-Based Prediction of Oroantral Communication after Tooth Extraction Utilizing Preoperative Panoramic Radiography
Source: Diagnostics (Basel). 2022 Jun 6;12(6):1406. doi: 10.3390/diagnostics12061406 (PMC9221677; doi:10.3390/diagnostics12061406)
Supplement: Supplementary file 1 [file diagnostics-12-01406-s001.zip › Supplementary file 1.pdf]

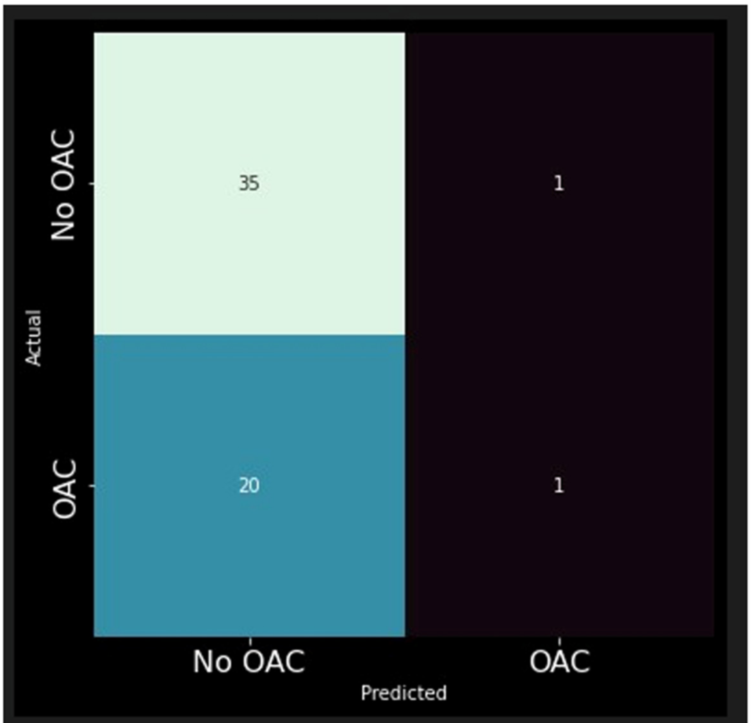

	precision	recall	f1-score	support
0	0.64	0.97	0.77	36
1	0.50	0.05	0.09	21
accuracy			0.63	57
macro avg	0.57	0.51	0.43	57
weighted avg	0.59	0.63	0.52	57

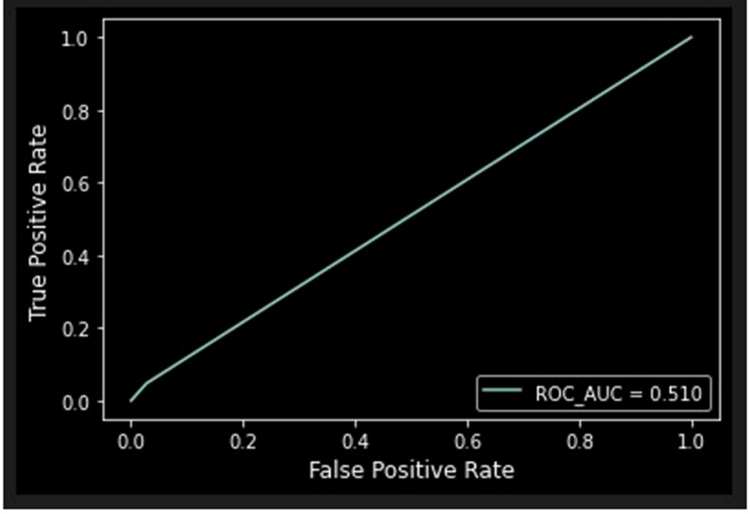

Figure S1. Confusion matrix, model performance measures, and receiver operating characteristic (ROC) curve for the EfficientNet algorithm. Precision: TP/(TP+FP); Recall: TP/(TP+FN); F1 score: 2*(recall*precision)/(recall + precision); support: actual occurrence of the class in the dataset. Values are showing the metrics for the independent test dataset (hold-out dataset).

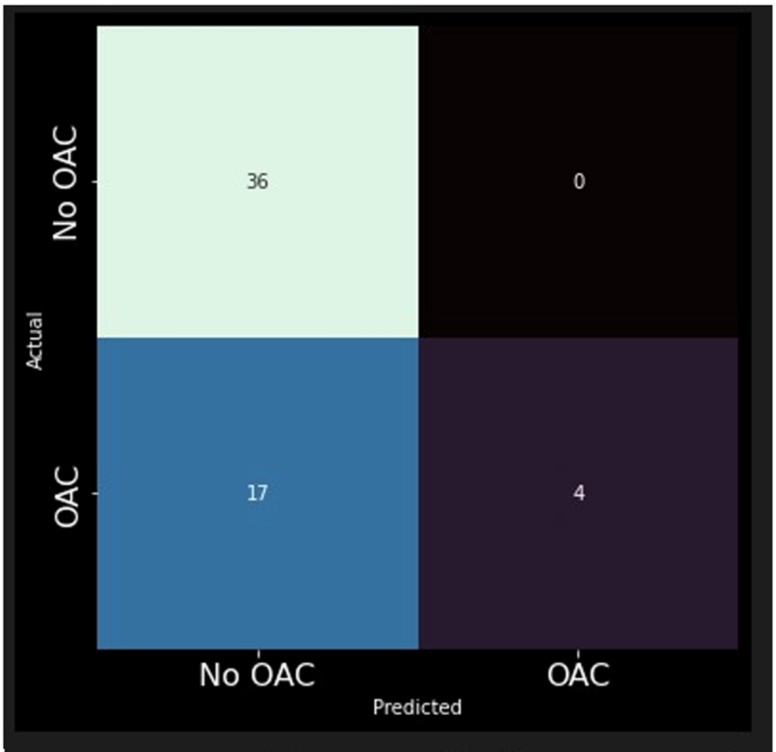

	precision	recall	f1-score	support
0	0.68	1.00	0.81	36
1	1.00	0.19	0.32	21
accuracy			0.70	57
macro avg	0.84	0.60	0.56	57
weighted avg	0.80	0.70	0.63	57

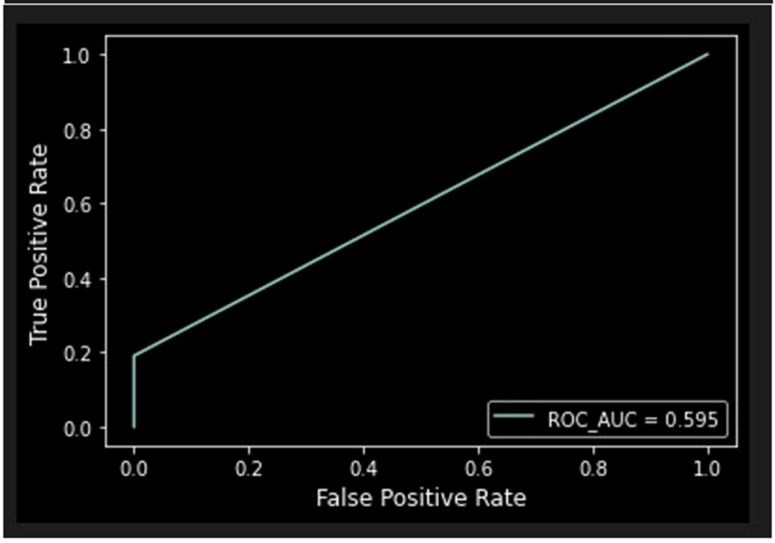

Figure S2. Confusion matrix, model performance measures and receiver operating characteristic (ROC) curve for the InceptionV3 algorithm. Precision: TP/(TP+FP); Recall: TP/(TP+FN); F1 score: 2*(recall*precision)/(recall + precision); support: actual occurrence of the class in the dataset. Values are showing the metrics for the independent test dataset (hold-out dataset).

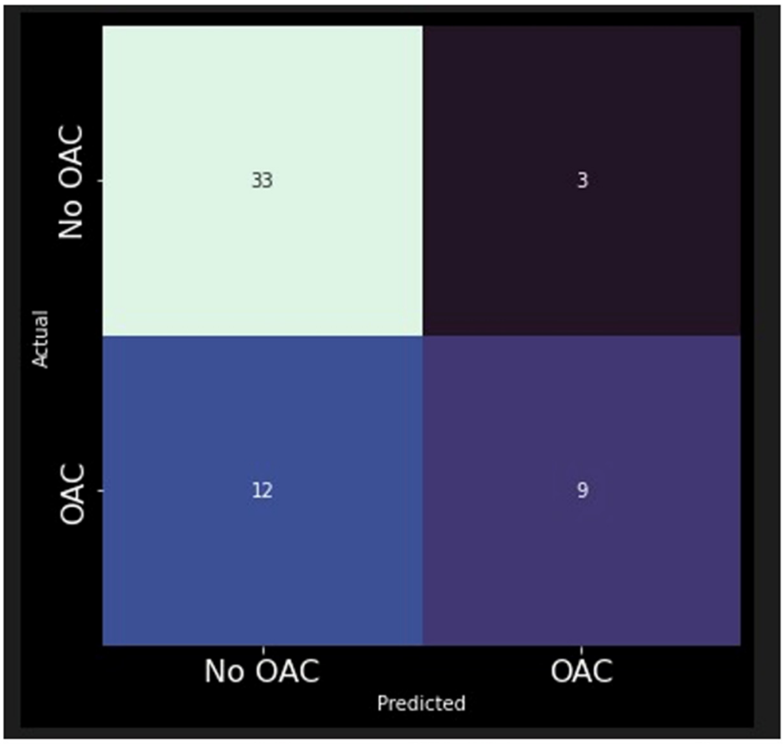

	precision	recall	f1-score	support
0	0.73	0.92	0.81	36
1	0.75	0.43	0.55	21
accuracy			0.74	57
macro avg	0.74	0.67	0.68	57
weighted avg	0.74	0.74	0.72	57

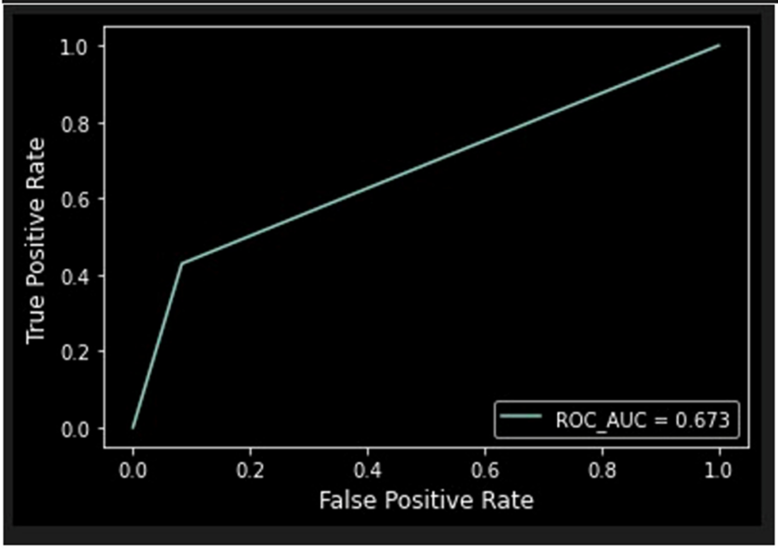

Figure S3. Confusion matrix, model performance measures and receiver operating characteristic (ROC) curve for the MobileNetV2 algorithm. Precision: TP/(TP+FP); Recall: TP/(TP+FN); F1 score: 2*(recall*precision)/(recall + precision); support: actual occurrence of the class in the dataset. Values are showing the metrics for the independent test dataset (hold-out dataset).

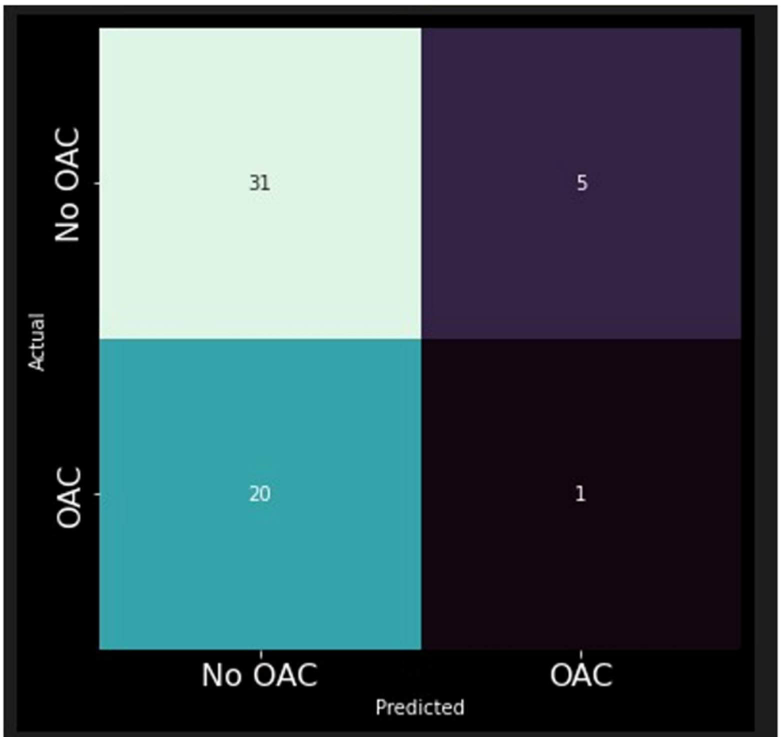

	precision	recall	f1-score	support
0	0.61	0.86	0.71	36
1	0.17	0.05	0.07	21
accuracy			0.56	57
macro avg	0.39	0.45	0.39	57
weighted avg	0.45	0.56	0.48	57

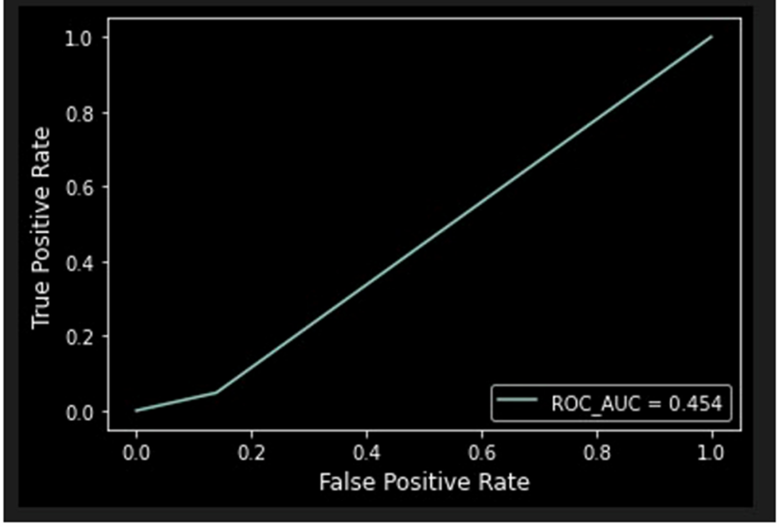

Figure S4. Confusion matrix, model performance measures and receiver operating characteristic (ROC) curve for the ResNet50 algorithm. Precision: TP/(TP+FP); Recall: TP/(TP+FN); F1 score: 2*(recall*precision)/(recall + precision); support: actual occurrence of the class in the dataset. Values are showing the metrics for the independent test dataset (hold-out dataset).

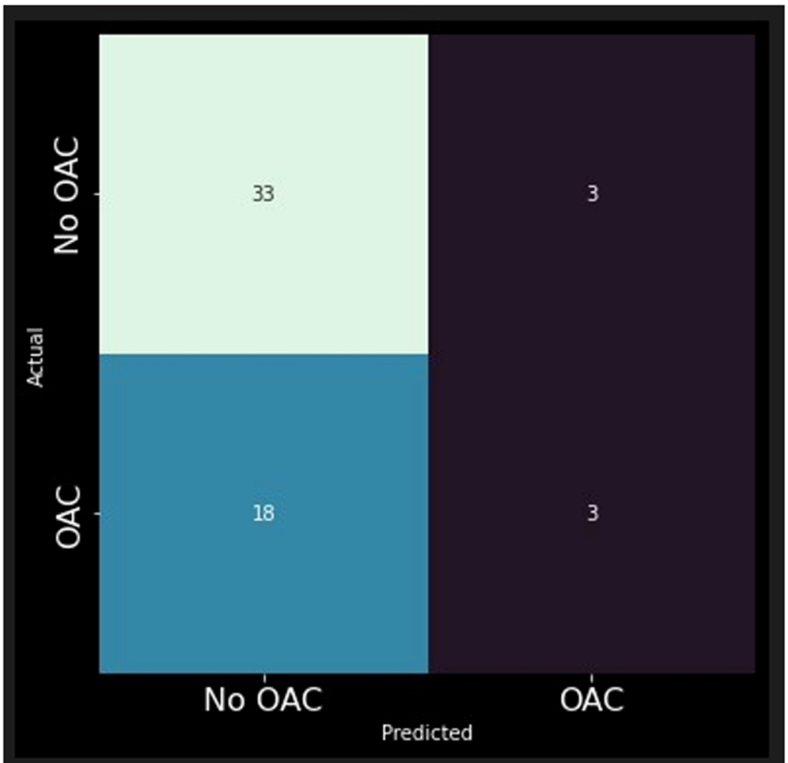

	precision	recall	f1-score	support
0	0.65	0.92	0.76	36
1	0.50	0.14	0.22	21
accuracy			0.63	57
macro avg	0.57	0.53	0.49	57
weighted avg	0.59	0.63	0.56	57

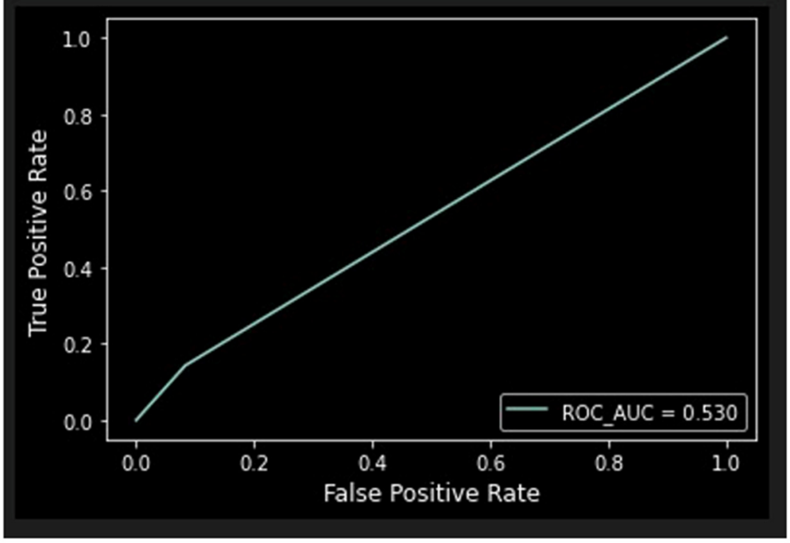

Figure S5. Confusion matrix, model performance measures, and receiver operating characteristic (ROC) curve for the VGG16 algorithm. Precision: TP/(TP+FP); Recall: TP/(TP+FN); F1 score: 2*(recall*precision)/(recall + precision); support: actual occurrence of the class in the dataset. Values are showing the metrics for the independent test dataset (hold-out dataset).
