# Supplementary figures and images for: Artificial Intelligence-Based Prediction of Oroantral Communication after Tooth Extraction Utilizing Preoperative Panoramic Radiography
Source: Diagnostics (Basel). 2022 Jun 6;12(6):1406. doi: 10.3390/diagnostics12061406 (PMC9221677; doi:10.3390/diagnostics12061406)

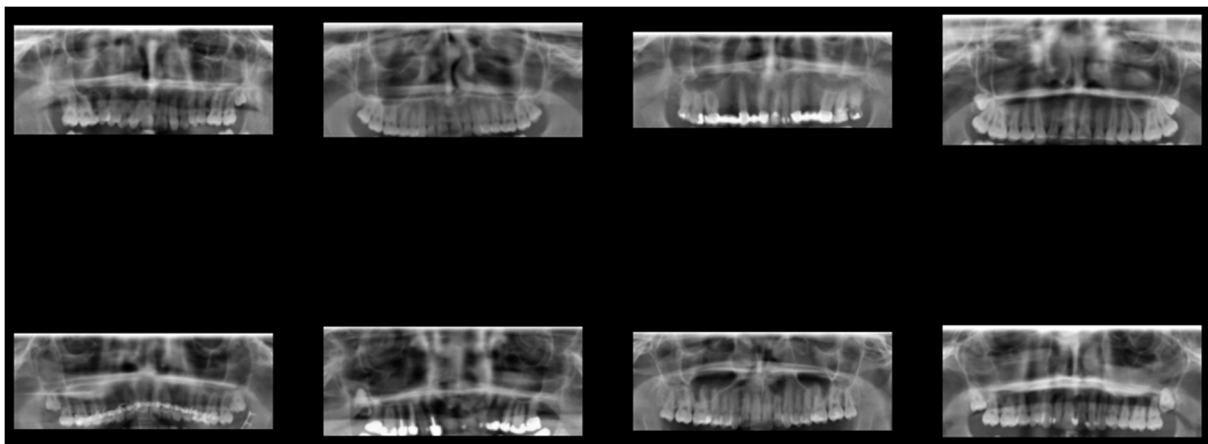

**Figure S6.** Example images from the whole data set.

Supplement: Supplementary file 1 [file diagnostics-12-01406-s001.zip › Supplementary file 2.pdf]
